# Individual differences in local functional brain connectivity affect TMS effects on behavior

**DOI:** 10.1038/s41598-020-67162-8

**Published:** 2020-06-26

**Authors:** Carsten Gießing, Mohsen Alavash, Christoph S. Herrmann, Claus C. Hilgetag, Christiane M. Thiel

**Affiliations:** 1 Biological Psychology Lab, Department of Psychology, School of Medicine and Health Sciences, Research Center Neurosensory Science and Systems, Carl von Ossietzky University Oldenburg, 26111 Oldenburg, Germany; 2 Department of Psychology, University of Lübeck, 23562 Lübeck, Germany; 3 Experimental Psychology Lab, Department of Psychology, School of Medicine and Health Sciences, Research Center Neurosensory Science and Systems, Carl von Ossietzky University Oldenburg, 26111 Oldenburg, Germany; 4 Cluster of Excellence Hearing4All, 26111 Oldenburg, Germany; 5 Institute of Computational Neuroscience, University Medical Center Hamburg-Eppendorf, 20246 Hamburg, Germany

**Keywords:** Attention, Functional magnetic resonance imaging, Psychology, Transcranial magnetic stimulation

## Abstract

Behavioral effects of transcranial magnetic stimulation (TMS) often show substantial differences between subjects. One factor that might contribute to these inter-individual differences is the interaction of current brain states with the effects of local brain network perturbation. The aim of the current study was to identify brain regions whose connectivity before and following right parietal perturbation affects individual behavioral effects during a visuospatial target detection task. 20 subjects participated in an fMRI experiment where their brain hemodynamic response was measured during resting state, and then during a visuospatial target detection task following 1 Hz rTMS and sham stimulation. To select a parsimonious set of associated brain regions, an elastic net analysis was used in combination with a whole-brain voxel-wise functional connectivity analysis. TMS-induced changes in accuracy were significantly correlated with the pattern of functional connectivity during the task state following TMS. The functional connectivity of the left superior temporal, angular, and precentral gyri was identified as key explanatory variable for the individual behavioral TMS effects. Our results suggest that the brain must reach an appropriate state in which right parietal TMS can induce improvements in visual target detection. The ability to reach this state appears to vary between individuals.

## Introduction

There is a growing interest in understanding how behavioral effects of local brain perturbations, such as those produced by transcranial magnetic stimulation (TMS), are modulated by large-scale brain network states^1–3^. Several studies documented that behavioral performance following brain stimulation considerably varies between individuals, depending upon factors such as hormone level, genetics, or ongoing cortical activity^4–7^. At the neural level, focal electrical or magnetic brain stimulation was shown to induce changes in widely-distributed functional brain networks, rather than just to produce local ‘virtual lesions’^8–10^. Remote changes in brain networks result from spread of neural activity, as well as from interactive processes in which effects of local brain perturbations are compensated by a far-reaching re-organization of current network states^4,11,12^. Accordingly, it is plausible to assume that inter-individual differences of distributed brain networks before and following TMS contribute to the inter-individual behavioral variability of TMS effects^13^.

**Figure 1:**
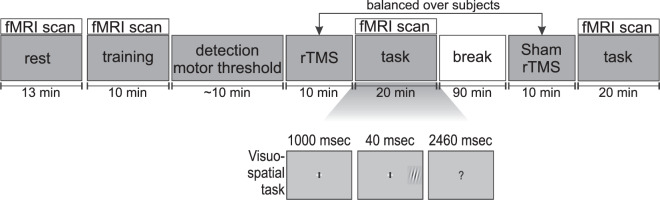
Experimental design and task. Following a resting state measurement, training, and an assessment of TMS motor threshold, participants received either repetitive transcranial magnetic stimulation (rTMS) or sham stimulation on the posterior parietal cortex, counterbalanced across subjects. The stimulation was followed by performing a visuospatial detection task inside the MRI scanner. Subjects were instructed to detect small Gabor patches that were presented either on the left, right, or both sides of the peripheral visual field. Following a break of 90 minutes and a period of, alternatively, TMS or sham stimulation, participants performed the task for the second time.

In the present study, young healthy participants underwent an fMRI experiment combined with TMS in order to assess brain network states before and after brain stimulation. After an initial resting state measurement, the participants performed a visuospatial target detection task with left, right and bilateral targets, once after posterior parietal TMS and once after sham stimulation (see Fig. 1). The main question was whether the individual participants’ brain network state before and following TMS stimulation could explain inter-individual differences in parietal TMS effects on attention. To address this question, we focused on one of the most fundamental measures of network integration, functional connectivity, and investigated inter-individual differences in functional connectivity using a whole-brain voxel-wise analysis. We then related the individual functional connectivity before and following TMS to the individual behavioral TMS effects.

Previous behavioral studies suggest that TMS of the right parietal cortex impairs visual spatial attention^14–16^, partly based on the distortion of the balance of interhemispheric inhibition^17,18^. In agreement with these results, TMS on left parietal cortex showed reversed patterns of brain activations within the left and right hemisphere following TMS^19^. In contrast to previous studies, the current analyses investigated the interactions of right parietal cortex stimulation and whole brain functional connectivity, rather than BOLD activation. Moreover, we separated intra- and interhemispheric connectivity and analysed the contribution of connectivity within and between both brain hemispheres towards individual attentional TMS effects^20^.

Furthermore, we used an elastic net analysis, a variable selection approach from machine learning, to identify the most informative set of brain regions associated with behavioral TMS effects^21^. Thereby, our method adaptively created a model between two extremes that captured how explanatory information was distributed across the brain. The model adapts towards one end of the dimension if explanatory information is broadly integrated over large parts of the brain. It adapts towards the other end of the dimension if only few brain regions are needed to receive the best explanatory model. In summary, the current analyses investigated large-scale connectivity before and following right parietal TMS to identify a set of brain regions whose intra- and/or cross hemispheric connectivity best explains individual TMS effects.

## Method

### Subjects

Twenty-two right handed volunteers (all Caucasians; 16 females, 6 males; age range: 18–31 years, mean: 24 years) with no reported history of neurological or psychiatric diseases gave informed consent to participate in the study. We excluded two subjects who showed large scan-to-scan movements (more than 3 mm voxel size) or a target detection lower than chance level. The remaining 20 subjects had normal or corrected to normal vision. A clinical evaluation was first carried out to ensure that subjects had no conditions contraindicative for TMS stimulation. Ethics approval was obtained from the local ethics committee at the Carl von Ossietzky University of Oldenburg (Germany, Commission for Research Impact Assessment and Ethics). All procedures were carried out with written informed consent of all subjects, in accordance with the principles of the Declaration of Helsinki, and in accordance with the relevant guidelines and regulations. Subjects received a monetary compensation for participation.

### Experimental design and task

Each experimental session was divided into five parts (see Fig. 1): (1) a resting-state measurement followed by a training of the task inside the scanner, (2) a motor threshold (MT) measurement followed by a first stimulation outside the scanner, (3) a first fMRI-task scan, (4) a second stimulation, and (5) a second fMRI-task scan. There was a break for 90 minutes after the first fMRI-task (i.e., third part) and before the second stimulation (i.e., fourth part). Preparation of a participant for imaging after stimulation took on average five minutes (±0.51 minutes SEM, from the end of stimulation until the initiation of task performance inside the scanner). Half of the subjects received real stimulation first. Structural images were acquired at the end of the session. During the resting-state scan, subjects were instructed to lie still inside the scanner, not to think about anything specific for around 13 minutes. To avoid brain activity related to involuntary eye movements, subjects were asked to close their eyes^22^. During the visuospatial task, adapted from Hilgetag, *et al*.^17^ and used in Alavash, *et al*.^23^, subjects were asked to report the location of small Gabor patches briefly presented either in the left, or right, or both the left and right (bilateral) visual periphery on a grey background. Subjects used their right hand to report the targets. They used the index or ring finger to report left or right targets, respectively. The middle finger was used to indicate the bilateral stimulus condition. Before performing the actual task and following the resting-state scan, the participants performed a short training inside the scanner. The duration of the training was approximately 10 minutes.

Each trial started by presenting a fixation cross for 1000 ms. Following the fixation cross, a peripheral unilateral or bilateral target appeared for 40 ms at approximately 20 degrees of visual angle. There were also trials on which no target was presented (catch trials). Each trial ended by changing the fixation cross into a question mark, asking the subject to manually report the target location as quickly and accurately as possible. The total duration of each trial was 3.5 seconds. In addition, we included ‘button press’ conditions. On these trials, either the left or right ‘wing’ of the fixation cross changed into black, indicating which button the participant should press. For each unilateral and bilateral condition, the contrast of the Gabor patch was fixed at two levels, one easy (standard deviation of pixel intensities = 0.026) and one difficult (standard deviation of pixel intensities = 0.012), to avoid ceiling or floor effects. There were 32 trials per easy or difficult target stimulus for each unilateral and bilateral condition. The duration of the visuospatial task was approximately 20 minutes (781 functional volumes). The different trial types were equally distributed across the four different task blocks so that within each task block there were all trial types (82 trials per block including 48 trials with visual target, 10 ‘button press’ trials, and 24 ‘null’ trials in which only a fixation cross with grey wings was presented; 4 × 82 = 328 trials during each fMRI-task scan).

### TMS protocol

Before TMS, the individual participants’ motor threshold (MT) was assessed relative to the maximum output of the stimulator (MAG Power, Brain Products). The resting motor threshold was defined as the lowest stimulation intensity that elicited at least five finger twitches in response to ten consecutive single-pulses delivered to the motor hot spot.

Since previous studies did not find a significant correlation between motor threshold and neural activations within non-motor brain areas^24^, we used a fixed TMS stimulation intensity instead of a percentage of motor threshold. During the main repetitive stimulation, similar to previous studies, the stimulation intensity was fixed at 40 percent relative to the maximum output of the stimulator^25^. As a safety criterion, it was verified that this stimulation intensity was less than 90 percent of individuals’ motor threshold^26^. During the stimulation period, 1 Hz repetitive TMS pulses were applied to the right parietal cortex (P4 electrode site according to the EEG 10–20 standard) for a duration of 10 min. Okamoto, *et al*.^27^ documented that, averaged across subjects, a coil position on P4 showed the smallest distance to a cortex surface of the right angular gyrus with the MNI coordinates x = 37, y = −75, z = 49 (with a standard deviation of 7.6 mm; see Fig. 3a, plot 1). Pulses were biphasic with the polarity of the first half being always positive. A figure-of-eight coil (196 × 100 mm; inner diameter: 20 mm, outer diameter: 95 mm) with a lateral-medial coil current direction was used. Sham TMS was administered by tilting the coil 90 degrees off the scalp, with one wing of the coil touching the scalp.

### MRI acquisition

A Siemens MAGNETOM Verio MRI system (3 T, Erlangen, Germany) with a 12 channel head coil was used to obtain T2*-weighted echo-planar images with BOLD contrast. A total of 527 volumes during resting state, 407 volumes during training, and 781 volumes during task performance of 27, 3.5-mm-thick axial slices were acquired. Scanning was performed sequentially (ascending) with a between-slice gap of 0.87 mm, repetition time of 1.5 s, echo time of 30 ms, flip angle 80°, matrix size of 64 × 64, and voxel size of 3 × 3 × 3.5 mm^3^.

### MR data preprocessing

After removing the first nine images, to account for possible T1 saturation effects, the functional images were corrected for head motion by spatially realigning them to the first volume using SPM8. Then, the functional images were spatially normalized to standard stereotaxic MNI space. To avoid anatomical misalignment, to remove high BOLD signal variations, and to increase the signal-to-noise ratio data were smoothed with an 8 mm Gaussian kernel^28,29^. Following preprocessing (realignment, normalization, smoothing) for each subject and TMS condition, BOLD time series were extracted in 112,495 voxels, in which BOLD time courses were consistently measured across subjects. In order to minimize the effects of spurious temporal correlations induced by physiological and movement artifacts, a GLM was used to regress out the six rigid-body movement parameters, four white-matter as well as three cerebrospinal fluid (CSF) mean time series^30,31^. In addition, voxel time series were corrected for linear trends. It was previously shown that behavioral correlates of brain network topology are best observed in low-frequency functional brain networks^32–35^. Therefore, regional time series were band-pass filtered with a Chebyshev Type 1 filter of order 8 within the range of 0.01–0.1 Hz^36,37^. To avoid spurious negative correlations, no global signal regression was performed^38,39^.

### Elastic net analysis: Identification of brain regions associated with behavioral TMS effects

Using an elastic net analysis, we investigated whether the mean functional connectivity across the entire brain either during sham or TMS condition, or a change in functional connectivity following TMS (TMS minus Sham) explained the behavioral TMS effects. The analysis focused on the analysis of TMS-induced changes in accuracy in bilateral trials, the condition that can be expected to show the strongest TMS effect^17^.

#### Connectivity matrices

In a first step, voxel-wise mean functional connectivity maps were computed by calculating Fisher’s z-transformed Pearson’s linear correlations between all pairs of 112,495 voxel BOLD time series (see above). Negative correlations were deleted to avoid spurious negative associations possibly induced by the removal of white matter, ventricle and head motion time courses in the previous pre-processing steps^40^. The mean functional connectivity of these voxels was calculated for resting state, sham and TMS conditions. Theses analyses were separately performed for intra-hemispheric and cross-hemispheric functional connections since, according to the interhemispheric competition model, attentional spatial biases might result from a change in interhemispheric inhibition and intra-hemispheric integration^17^. Each pair of voxels was grouped into intra- or cross-hemispheric connectivity by a normalized version of the brain template of Shen, *et al*.^41^, and for each voxel the mean functional connectivity values for intra-hemispheric and cross-hemispheric functional connections were computed. Thus, the analysis of mean intra-hemispheric and cross-hemispheric functional connectivity was still performed in the voxel space.

#### Preselection of variables

To develop a valid association model with a manageable set of regressors, only those brain regions were included as regressors that were expected to be influenced by TMS. Thus, only brain regions were selected that showed a minimum functional connectivity with a specific region within the right angular gyrus (MNI coordinates x = 37, y = −75, z = 49). According to Okamoto, *et al*.^27^, this brain regions is the average TMS side on the cortex surface if P4 is stimulated by TMS (see section 2.3). To identify brain regions connected with this voxel position, we used the tools provided by Neurosynth.org (https://neurosynth.org/locations/; 13^th^ of March 2020). The analysis was performed on an independent data set of 1000 subjects including data from the Brain Genomics Superstruct Project (https://dataverse.harvard.edu/dataverse/GSP; 13th of March 2020) and others^42,43^. Based on this data set, the functional connectivity between each voxel and the voxel’s time series in the right angular gyrus (MNI coordinates x = 37, y = −75, z = 49) was computed. Only brain regions were included in the following analyses that showed an absolute Pearson’s correlation of at least 0.1. This relatively liberal threshold was used in order to keep the number of regressors large enough to prevent useful regressors from being left out^44^.

To further reduce noise effects, within each distribution of intra-hemispheric and cross-hemispheric links, only regressors entered into the subsequent analysis whose mean connectivity averaged across subjects belonged to the top 80 percent, leaving 32,739 regressors for intra- and cross-hemispheric connectivity, respectively.

#### Elastic-Net Algorithm

The aim of the current study was to identify the most informative set of intra- and interhemispheric connections to explain behavioral TMS effects. Within the machine learning literature, different regularized regression methods have been suggested in case that the number of regressors *p* (2*32,739) exceeds the number of predicted values (in our case, the number of subjects, N = 20). In the current approach, an elastic net analysis was used which estimates the regression weights *β* und constant term *β*_0_ by minimizing the following loss function^21,45^

1 $$\mathop{\min }\limits_{({\beta }_{0},\beta )\in {R}^{p+1}}{R}_{{\rm{\lambda }}}({\beta }_{0},\beta )=\mathop{{\rm{\min }}}\limits_{({\beta }_{0},\beta )\in {R}^{p+1}}\left[\frac{1}{2N}\mathop{\sum }\limits_{i=1}^{N}{({y}_{i}-{\beta }_{0}-{x}_{i}^{T}\beta )}^{2}+\lambda {P}_{\propto }(\beta )\right],$$

where

2 $${P}_{\propto }(\beta )=(1-\alpha )\frac{1}{2}\Vert \beta {\Vert }_{{\ell }_{2}}^{2}+\alpha {\Vert \beta \Vert }_{{\ell }_{1}}$$

3 $$=\,\mathop{\sum }\limits_{j=1}^{p}\left[\frac{1}{2}(1-\alpha ){\beta }_{j}^{2}+\alpha |{\beta }_{j}|\right]$$

Thereby let *N* be the number of subjects, *y*_*i*_ the mean-corrected response variable (in our case the behavioral TMS effect of subject *i*), and *x*_*i*_ the vector of *p* standardized regressor variables of subject i (in our case the functional connectivity; see 21, p. 303 for further details).

In contrast to multiple regression, which minimizes the sum of squares of the residuals, in regularized regression the loss function includes also a penalty term P_α_ (see Eq. 1). Here, P_α_, the elastic net penalty, is a compromise between the penalties of two different regularized regression methods, the penalties of ridge-regression and the lasso method see^45^. Whereas λ controls the general strength of the penalty P_α_, α is the mixing parameter that controls the relative contribution from the lasso ℓ1 norm penalty (alpha = 1) and the ridge ℓ2 norm (alpha = 0) penalty (see Eqs. 2 and 3). According to Zou and Zhang^46^, the ℓ1 penalty of the Elastic-Net performs automatic variable selection, whereas the ℓ2 penalty stabilizes the solution paths and hence improves the prediction. The performance of ridge regression and lasso depends on the statistical properties of the data. From a Bayesian perspective, ridge regression is ideal in case of many regression coefficients with non-zero elements (drawn from a Gaussian distribution); in contrast, lasso performs best in case of a Laplace prior with only few non-zero coefficients and many zero coefficients^45^.

#### Mixing of ridge regression and lasso

By changing the mixing parameter α, the functional connectivity state of widely distributed brain networks or, by contrast, the connectivity of only a few local brain regions was incorporated into the model. To find the best association model, we adapted functions from the R-statistic toolbox ‘biglasso’ (https://arxiv.org/abs/1701.05936) and tested a sequence of 100 λ values, starting with 0.01 and ending with 0.61 equally spaced on the log scale^47^. For α, we choose nine equally spaced steps, starting with 0.1 and ending with 0.9, and 0.99 to adequately sample the parameter space (see Results section). To describe the significance of the models, we computed the proportion of variance R^2^ explained by each model, as estimated by leave-one subject-out cross-validation. In addition, using non-parametric one-sided permutation tests with 100,000 iterations, it was tested for each α if the optimal model significantly reduced the prediction error in comparison to an intercept only model^48^. The prediction errors of the tested model and the reduced intercept-only-model was estimated by leave-one subject-out cross-validations and randomized during permutation testing. Furthermore, we analyzed the consistency of the selected regressors across all significant models to show the stability of the results.

### Statistical analysis of behavioral measures

Repeated measures analysis of variance (ANOVA) models were used to estimate the main effects of TMS (sham vs. TMS stimulation), time-on-task (four different task blocks), stimulus condition (left, right, bilateral targets), stimulus difficulty (high vs. low contrast level of Gabor patches) and their interactions, for the accuracy of behavioral responses. For significance testing of Pearson’s correlations, two-sided, non-parametric permutation tests with 10,000 iterations were used in which the to be correlated variables were randomly paired. Reaction times were not analyzed since stimuli were indicated with different fingers which often induces artificially increased response times of the middle finger^49^.

### Effects of individual differences in motor threshold and head motion

Analysis of covariance (ANCOVA) was computed to investigate whether the association between ‘mean functional connectivity’ and behavioral TMS effects was biased by individual differences in motor threshold. The model included the ‘individual motor threshold’ as first factor, ‘mean functional connectivity’ as second factor, and their interaction as third factor. Using type I sums of squares, it was tested whether each factor led to an incremental improvement in error reduction. A significant result of the factor ‘mean functional connectivity’ would document that the mean functional connectivity is significantly associated with the TMS effects on accuracy over and above possible effects of individual differences in motor threshold.

Data censoring was not performed in order to avoid variations in the remaining number of measured fMRI data points which can impact inter-individual differences in functional connectivity^50^. However, to control possible effects of head movements on group level, the same ANCOVA model was computed with individual average motion estimates as nuisance regressor instead of the ‘individual motor threshold’ (see above). As indicator for individual average head motion the mean framewise displacement was calculated for each subject^51,52^.

### Effects of TMS administration order

Similar to the ANCOVA reported above, the effect of TMS administration order on the linear association between functional connectivity and TMS-induced changes in the accuracy of bilateral targets was analyzed. The ANCOVA included the ‘order of TMS administration’ as first factor, the ‘mean functional connectivity’ as second factor, and their interaction as third factor. Following the same testing procedure as above, for each factor the incremental improvement in error reduction (type I sums of squares) was investigated.

## Results

### Visuospatial target detection and bias

**Figure 2:**
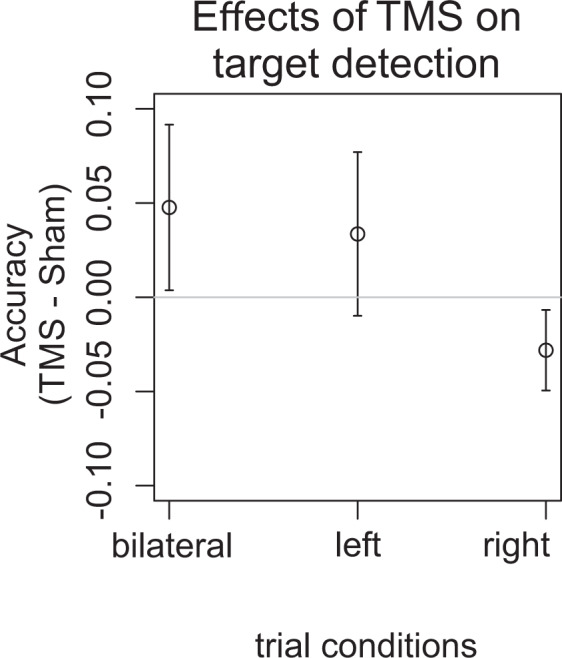
Effects of posterior parietal repetitive transcranial magnetic stimulation (TMS) on visual target detection. Mean TMS effects (TMS minus Sham) on the accuracy during bilateral, left and right target detection trials are shown with standard error of means (SEMs). The strongest behavioral improvements of TMS on target detection were found for bilateral trials and were weaker for unilateral targets presented in the left or right visual hemi-field.

Participants were able to perform the task and reached an average accuracy of 0.54 ± 0.03 SEM averaged across left, right and bilateral trials pooled over TMS and sham stimulation. The accuracy of bilateral trials only was 0.48 ± 0.04 SEM. The statistical analysis of the behavioral data revealed a significant ‘stimulus condition’ by ‘TMS’ interaction effect: the TMS effect was significantly different for left, right, and bilateral targets, with larger TMS induced increases in accuracy within bilateral than right trials (F(2,38) = 3.55,p < 0.05; see Fig. 2). The data also revealed a significant main effect of time-on-task (F(3,57) = 29.16,p < 0.001), stimulus condition (F(2,38) = 25.2,p < 0.001), and stimulus difficulty (F(1,19) = 455.9,p < 0.001), but no further main effects or interactions with the TMS condition. Means of behavioral accuracies and standard errors of means (SEMs) within the different TMS and trial conditions are shown in Table 1.

**Table 1:** Means of behavioral accuracies and standard errors of means (SEMs) within TMS and trial conditions.

		Trial condition		
		Bilateral	Left	Right
TMS condition	TMS	0.50 ± 0.05	0.49 ± 0.05	0.65 ± 0.04
	Sham	0.45 ± 0.05	0.46 ± 0.04	0.68 ± 0.03

mean ± SEM.

### Post-TMS functional connectivity explains behavioral TMS effects

#### Significance and test of model consistency

**Figure 3:**
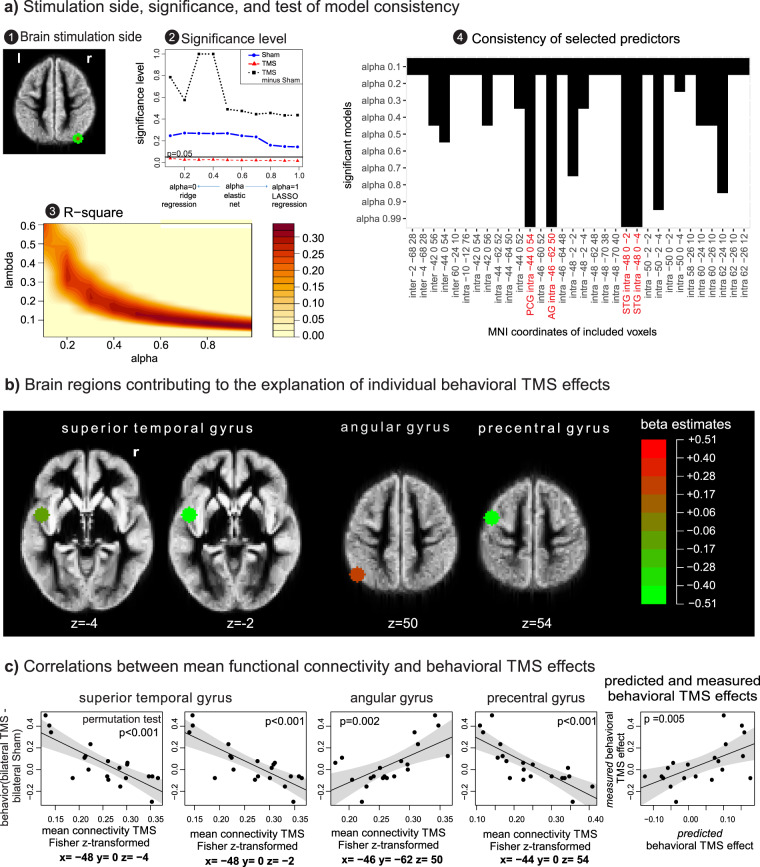
Connectivity states associated with behavioral TMS effects. An elastic net analysis was performed to explain the variation in behavioral TMS effects by the voxel-wise mean connectivity measured following (i) Sham, (ii) TMS stimulation, and (iii) by the change of mean connectivity following TMS. **(a) Stimulation side, significance, and test of model consistency**. *Plot 1*: The position of the TMS coil on P4 showed the smallest average distance to the right angular gyrus. *Plot 2*: Mean functional connectivity following TMS showed a significant association with behavioral TMS effects. Data points below the black horizontal line indicate significant p-values (p < 0.05). *Plot 3:* Proportion of variance explained by the model, as estimated by cross-validation as function of alpha and lambda. Models with a large relative contribution from the lasso ℓ1 norm penalty (large alpha), and therefore only few regressor variables, tended to show better associations. *Plot 4:* For each of the ten significant models, brain regions that received non-zero regression coefficients and contributed to the model are graphically illustrated by black bars. All significant models include the same four regressor variables with non-zero coefficients that were also selected for the model with the best significance level. The corresponding voxel positions are presented in MNI coordinates. STG: superior temporal gyrus, AG: angular gyrus, PCG: precentral gyrus, inter: *inter*hemispheric connectivity, intra: *intra*hemispheric connectivity. **(b) Brain regions contributing to the explanation of individual behavioral TMS effects**. The anatomical positions of the four selected regressors included within the model with the smallest p-value are illustrated. For each of these brain regions, only the mean intra-hemispheric functional connectivity (and not the inter-hemispheric connectivity) was selected as regressor. The size of the estimated regression coefficients is color-coded. Voxel sizes of the explanatory brain regions were increased for illustrative reasons and plotted on co-registered and normalized T1 group mean images. **(c) Correlations between mean functional connectivity and behavioral TMS effects**. The single correlation between mean functional connectivity following TMS and the behavioral TMS effects are illustrated for each brain region selected in the elastic net analysis. Right plot: The correlation between predicted and measured behavioral TMS effects is shown.

In a next step, we aimed to answer the question of whether brain connectivity before and following TMS was associated with TMS effects on behavior. Following TMS, changes in accuracy in the detection of bilateral trials differed substantially between subjects. Using an elastic net analysis, this between-subject behavioral variability could be significantly explained by the brain functional connectivity measured following TMS. The elastic net models significantly reduced the prediction error in comparison to an intercept-only model (p < 0.05, permutation tests with 100,000 iterations) and showed the largest explained variance with large alpha, the mixing parameter between the ℓ1 and ℓ2 penalty (Fig. 3a, plot 2 and 3). Thus, the elastic net analyses showed better results for models with a large contribution of the ℓ1 norm penalty that contained only few regressor variables. That means, the best approach to predict behavioral TMS effects was to focus on the mean functional connectivity of only few brain regions. The model with the smallest p-value explained 33 percent of the prediction error (alpha = 0.99, Fig. 3a, plot 3). For voxel-wise mean connectivity measured following sham stimulation and changes of mean connectivity following TMS (TMS minus Sham), we found no significant results.

If regressor variables are highly correlated, elastic net analyses with large alpha tend to select only few regressor variables of the set of correlated regressors. This effect leads to parsimonious, but sometimes also unstable statistical models^53–55^. To investigate the consistency of the proposed models, we analyzed which regressor variables were selected within all significant models (Fig. 3a, plot 4). The results revealed that the four regressor variables selected within the model with the smallest p-value were consistently chosen within all significant models. That is, the mean intrahemispheric functional connectivity following TMS of the left angular gyrus, two regions within the left superior temporal gyrus, and left precentral gyrus was significantly associated with the individual change in accuracy on bilateral trials when subjects received TMS (Fig. 3b). Within the original model before variable selection, for each voxel one regressor for intra-hemispheric and one for interhemispheric connectivity was included. Following variable selection, for each included region only the between-subject variability in mean intra-hemispheric, and not the inter-hemispheric connectivity was selected within the model with the smallest prediction error.

#### Correlations between mean functional connectivity and behavioral TMS effects

For each of the selected brain regions included in the elastic net model, we also investigated the Pearson’s correlations between the behavioral TMS effects on bilateral target detection and mean functional connectivity (Fig. 3c). We found that higher functional connectivity of the superior temporal gyrus (MNI coordinates x = −48, y = 0, z = −4, r = −0.79, p < 0.001; x = −48, y = 0, z = −2, r = −0.80, p < 0.001, permutation tests with 10,000 iterations) and the precentral gyrus (x = −44, y = 0, z = 54, r = −0.80, p < 0.001) was associated with smaller TMS-induced behavioral improvements. However, higher mean functional connectivity of the angular gyrus correlated with larger improvements in performance (x = −46, y = −62, z = 50, r = 0.65, p = 0.002). As expected, we also found a significant correlation between the predicted and measured behavioral TMS effects within the elastic net analysis (r = 0.59, p = 0.005).

#### Effects of individual motor thresholds

The results of our ANCOVA documented for all brain regions included in the elastic net model that ‘mean functional connectivity’ remained a significant explanatory variable for accuracy over and above possible effects of motor thresholds. Within each brain region ‘motor threshold’ and the interaction ‘motor threshold’ by ‘mean functional connectivity’ showed only non-significant results (see Supplement Tab. 1). In addition, an analysis in which individual motor thresholds were correlated with the TMS effects on accuracy within bilateral trials revealed non-significant results (Pearson’s r = 0.07, permutation test p = 0.77).

#### Effects of individual differences in head motion

The results of our ANCOVA documented for all selected brain regions that ‘mean functional connectivity’ remained a significant explanatory variable for accuracy over and above possible effects of individual differences in head motions. Within each brain region ‘mean framewise displacement’ and the interaction ‘mean framewise displacement’ by ‘mean functional connectivity’ showed only non-significant results (see Supplement Tab. 2). In addition, an analysis in which individual framewise displacement was correlated with the TMS effects on accuracy within bilateral trials revealed non-significant results (Pearson’s r = −0.21, permutation test p = 0.39).

#### Effects of TMS administration order

Additional ANCOVAs revealed that for all selected brain regions the linear association between mean functional connectivity following TMS and the TMS effects on the accuracy of bilateral trials showed a significant linear association over and above possible effects of the order of TMS administration. Effects of ‘TMS condition order’ and the interaction ‘TMS condition order’ by ‘TMS effect’ showed only non-significant results (see Supplement Tab. 3). In summary, for both groups (subjects who received either TMS or Sham first) regressions models with similar slopes and intercepts describe the association between functional connectivity and TMS effects on accuracy in bilateral trials.

#### Effects of indirect correlations

Our results showed significant correlations between mean nodal functional connectivity following TMS and differences in accuracy following TMS and Sham stimulation. However, the reported correlations might result from a chain of two links, the correlation between connectivity following TMS and accuracy following TMS, and between accuracy following TMS and TMS-induced changes in accuracy. However, if subjects’ accuracy levels following TMS were regressed out, the reported correlations between functional connectivity and changes in accuracy remained significant for all brain regions: Both brain regions within the superior temporal gyrus showed a partial correlation of r = −0.81 (p < 0.001), the precentral gyrus of r = −0.75 (p < 0.001), and the angular gyrus of r = 0.53 (p = 0.02). In summary, these results suggest that the reported correlations are not mediated by inter-subject differences in performance.

## Discussion

### Summary

Previous studies documented that behavioral and neural effects of non-invasive brain stimulation considerably vary across individuals^4–6^. To assess potential determinants of individual behavioral TMS effects, the current study investigated the link between brain functional connectivity during sham stimulation and actual TMS of the right parietal cortex during the performance of a visuospatial target detection task. An “elastic net” analysis revealed that the intra-hemispheric connectivity of just a few brain regions during task processing explained significant parts of inter-subject variation in behavioral TMS effects. Following TMS, the functional task connectivity of the left superior temporal gyrus, left angular gyrus, and left precentral gyrus showed significant correlations with behavioral TMS effects. In contrast, connectivity following sham and the change in functional connectivity following TMS (ie, the difference in functional connectivity between TMS and sham) showed only non-significant results. In summary, behavioral TMS effects were significantly associated with the current individual states of functional connectivity of distinct brain regions that were not directly stimulated. Depending on the current state of brain networks following TMS, parietal cortical perturbation led to significantly different individual changes in visuospatial target detection accuracy.

Our partial correlation analyses support the conclusion that inter-individual differences in connectivity following TMS are significantly associated with a behavioral modulation that is indeed induced by TMS. TMS-induced changes in accuracy are not independent from the individual accuracy levels. Therefore, the reported correlation might reflect an indirect, non-causal link due to an underlying chain of correlations between connectivity measured following TMS, accuracy following TMS, and TMS induced changes in ‘accuracy’. Our partial correlation analyses controlled individual accuracy as a possible mediator variable and ruled out this alternative explanation.

### Integration of local perturbations on brain system level: effects of brain states following TMS

Mechanistic models explaining effects of local brain perturbation on whole-brain functional connectivity are currently intensely discussed. According to a computational model proposed by Gollo, *et al*.^2^, effects of local brain perturbation significantly varied between brain regions depending on the stimulated node’s structural connectivity. While local inhibition increases functional connectivity in regions with low structural connectivity, it decreases functional connectivity in brain regions with high structural connectivity see also^56–59^. Several empirical studies supported this link between the inter-regional differences in structural brain connectivity and neural effects of TMS^60–64^.

Our elastic net analysis also revealed that the current state of connectivity following TMS, rather than changes in functional connectivity between TMS and sham, was most informative for the explanation of individuals’ behavioral TMS effects. Behavioral TMS effects appeared to be dominated by the inter-individual differences in the current state of functional connectivity following brain perturbation, rather than by the baseline connectivity during sham stimulation or the extent of TMS-induced shifts in network connectivity. Particularly, the current level of mean functional connectivity of the left angular gyrus, left superior temporal gyrus, and left precentral gyrus significantly contributed to the explanation of behavioral TMS effects. Whereas subjects with higher mean connectivity of the angular gyrus showed larger improvements in performance following TMS, higher mean connectivity of the superior temporal and precentral gyri was associated with smaller behavioral improvements or even worse performance following TMS.

Brain regions with coordinates close to our selected region within the left superior temporal gyrus were previously linked to attentional processing^65,66^, and seem to be related to externally, rather than internally directed processing modes^67^. Brain areas of the superior temporal gyrus close to the left insular cortex were also shown to be part of the salience network involved in the dynamic switching between the central executive and default-mode networks^68–70^. The left angular gyrus has been related to intrinsic fluctuations in sustained attention and distractor processing^71^ and age-related effects on selective attention^72^. These individual differences might interact with motor preparation and execution within the left precentral gyrus. Previous results suggest that the successful and correct motor response depends on the interplay between attentional and motor processing^73^. In summary, our analysis suggests that the interindividual variance in behavioral TMS effects were best explained by the functional connectivity of three brain areas, playing an important role in the interaction between attentional and motor processing.

### Methodological considerations

#### Elastic net

To explain the behavioral variance of TMS effects, we used an elastic net^21^. This approach selects the best mixing parameter α which controls the relative contribution from the ℓ1 and ℓ2 norm penalty (see Methods section). By varying the parameter α, different numbers of regressors (few or many) were included within the statistical model. With high consistency across the significant models, our analysis suggests that the functional connectivity of only a few, local brain regions was sufficient to explain a significant part of the inter-subject variance of the behavioral TMS effects^5^.

#### Differences in motor threshold, TMS administration order, and head movement

Previous studies often selected the individual TMS intensity relative to the estimated motor threshold. Within the current studies we fixed the stimulation intensity since (1) the motor threshold can only be estimated within range of uncertainty and (2) within non-motor brain regions previous studies did not find a significant correlation between motor threshold and neural activations^24^. Using several ANCOVAs we could replicate our main finding that functional connectivity of the left superior temporal, angular, and precentral gyri were associated with the behavioral effects of TMS, even if differences of individual motor thresholds were statistically controlled (see Supplement Tab. 1). Further ANOVAs showed also that the association between connectivity and TMS effects on performance in bilateral trials remained significant if the confounding effect of the TMS administration order (first sham or TMS) and individual differences in head movement was statistically reduced (see Supplement Tab. 2 and 3). Thus, the empirical analyses revealed no hints that individual differences in motor threshold, head movements, or the TMS administration order might have biased our findings.

#### Limitation of the analyzed sample size

The elastic net model was estimated with a relatively small sample size (N=20). Small sample sizes can lead unstable models and possible overfitting^74^. Within our analysis we identified a simple model with only four regressors. These four regressors were consistently included within the best performing models across different alpha levels (Fig. 3a, plot 4). Both points argue for the stability of our results and against overfitting. However, future studies with larger data sets are needed to support our current exploratory findings.

### Conclusion and future perspectives

Following TMS, the individual state of brain connectivity of only four brain regions, that are strongly involved in attentional and motor processing, explained a significant amount of individual variation in the change in accuracy on bilateral trials. Whether TMS has an impact on the accuracy appears to depend on the ability of subjects to reach an appropriate brain state for performing the task correctly. This ability seems to vary between subjects. Remarkably, intra-hemispheric connectivity seems to show stronger impact on behavioral TMS effects than inter-hemispheric connectivity. However, due to the relatively small sample size, the current results have to be interpreted with caution and need further support. Future studies might manipulate or access the functional connectivity of these brain regions to control between subject difference in non-invasive brain stimulation, to improve therapeutic effect of TMS, and to find the most appropriate intervention method for an individual patient with a certain brain network state^4,6,75–78^.

## Supplementary information

Supplementary information.

## Data Availability

The datasets analyzed during the current study are available from the corresponding author on reasonable request.
