# Supplementary material for: Individual differences in local functional brain connectivity affect TMS effects on behavior
Source: Sci Rep. 2020 Jun 26;10:10422. doi: 10.1038/s41598-020-67162-8 (PMC7320140; doi:10.1038/s41598-020-67162-8)
Supplement: Supplementary file 1 — Supplementary information. [file 41598_2020_67162_MOESM1_ESM.pdf]

Individual differences in local functional brain connectivity affect TMS effects on behavior

Carsten Gießing[1], Mohsen Alavash[1,2], Christoph S. Herrmann[3,4], Claus C. Hilgetag[5], Christiane M. Thiel[1,4]

[1]Biological Psychology Lab Department of Psychology, School of Medicine and Health Sciences, Research Center Neurosensory Science and Systems, Carl von Ossietzky University Oldenburg, 26111 Oldenburg, Germany

[2]Department of Psychology, University of Lübeck, 23562 Lübeck, Germany

[3]Experimental Psychology Lab Department of Psychology, School of Medicine and Health Sciences, Research Center Neurosensory Science and Systems, Carl von Ossietzky University Oldenburg, 26111 Oldenburg, Germany

[4]Cluster of Excellence Hearing4All, 26111 Oldenburg, Germany

[5]Institute of Computational Neuroscience, University Medical Center Hamburg-Eppendorf, 20246 Hamburg, Germany

Content

Inline Supplementary Fig. 1. Bain mask for stimulus selection: Brain regions that were functionally connected with the stimulated brain region. 2

Supplement Tab 1. Effects of "individual motor thresholds" on the correlation between functional connectivity and TMS induced changes in accuracy detecting bilateral trials. 4

Supplement Tab 2. Effects of "framewise displacement" (FD) as measure of head motion on the correlation between functional connectivity and TMS induced changes in accuracy detecting bilateral trials. 5

Supplement Tab. 3. Effects of "TMS administration order" on the correlation between functional connectivity and TMS induced changes in accuracy detecting bilateral trials. 6

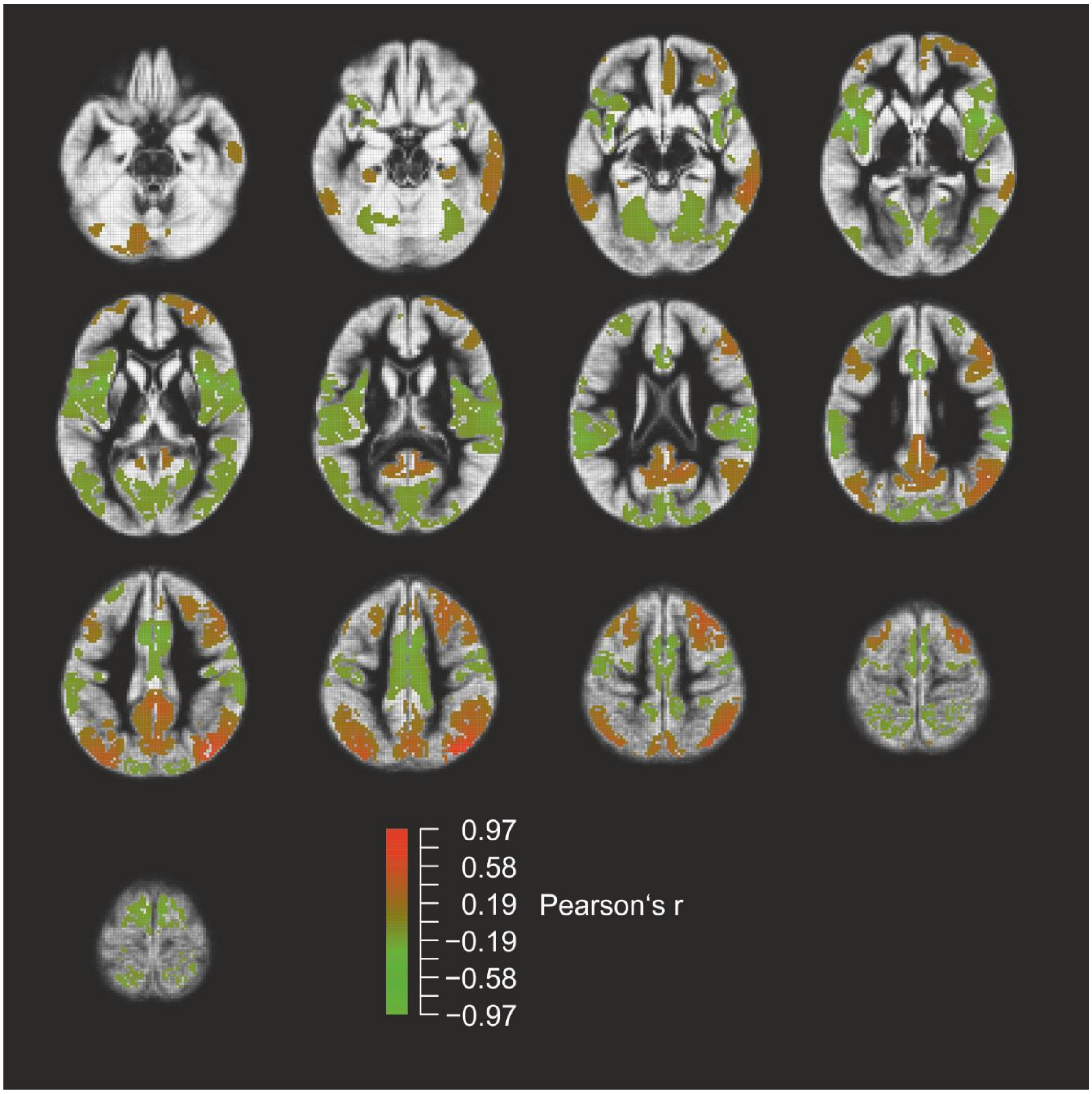

Inline Supplementary Fig. 1. *Bain mask for stimulus selection: Brain regions that were functionally connected with the stimulated brain region.*

To develop a valid prediction model, Burnham, et al. [1] suggests to selected predictors with high biologically plausibility, but to keep the number of predictors large enough to guard against omitting useful predictors. We assumed that brain regions, functionally connected with the stimulation side, significantly affect the behavioural effects of TMS. Thus, only those brain regions were analyzed that showed a minimum connectivity with the brain area stimulated by TMS within an independent data set. Okamoto, et al. [2] documented that, averaged across subjects, a coil position on P4 showed the smallest distance to a cortex surface of the right angular gyrus with the MNI coordinates x=37, y= -75, z= 49. Using the tools provided by Neurosynth.org (https://neurosynth.org/locations/; 13th of March 2020) we analyzed an independent data set of 1000 subjects including data from Thomas Yeo[3], Randy Buckner[4] and the Brain Genomics Superstruct Project (https://dataverse.harvard.edu/dataverse/GSP; 13th of March 2020). Based on these data set, brain regions were selected that showed a minimum

absolute Pearson's correlation of 0.1 with the time series of the average stimulation side. This relatively liberal threshold was used in order include a reasonable number of regressors (see above). While the selected brain regions with positive correlations are depicted in red, brain regions with negative correlations are shown in green.

Supplement Tab 1. *Effects of "individual motor thresholds" on the correlation between functional connectivity and TMS induced changes in accuracy detecting bilateral trials.*

l. superior temporal gyrus MNI: x=-48 y=0 z=-4			
	**Df**	**F value**	**Pr(>F)**
MT	1,16	0.236	0.633
functional connectivity	1,16	29.103	0.000
MT by functional connectivity	1,16	0.958	0.342

l. superior temporal gyrus MNI: x=-48 y=0 z=-2			
	**Df**	**F value**	**Pr(>F)**
MT	1,16	0.250	0.624
functional connectivity	1,16	31.641	0.000
MT by functional connectivity	1,16	1.153	0.299

l. angular gyrus MNI: x=-46 y=-62 z=50			
	**Df**	**F value**	**Pr(>F)**
MT	1,16	0.143	0.710
functional connectivity	1,16	11.702	0.004
MT by functional connectivity	1,16	0.224	0.642

l. precentral gyrus MNI: x=-44 y=0 z=54			
	**Df**	**F value**	**Pr(>F)**
MT	1,16	0.235	0.634
functional connectivity	1,16	29.585	0.000
MT by functional connectivity	1,16	0.248	0.625

An ANCOVA including the individual motor threshold as first factor, 'mean functional connectivity' as second factor, and their interaction as third factor were computed. Using type I sums of squares, it was tested whether each factor led to an incremental improvement in error reduction. The results documented for all brain regions that the factor "mean functional connectivity" was significantly associated with the accuracy over and above possible effects of motor threshold values.

Supplement Tab 2. *Effects of "framewise displacement" (FD) as measure of head motion on the correlation between functional connectivity and TMS induced changes in accuracy detecting bilateral trials.*

l. superior temporal gyrus MNI: x=-48 y=0 z=-4			
	**Df**	**F value**	**Pr(>F)**
FD	1,16	1.841	0.194
functional connectivity	1,16	25.714	0.000
FD by functional connectivity	1,16	0.198	0.663

l. superior temporal gyrus MNI: x=-48 y=0 z=-2			
	**Df**	**F value**	**Pr(>F)**
FD	1,16	1.940	0.183
functional connectivity	1,16	27.872	0.000
FD by functional connectivity	1,16	0.304	0.589

l. angular gyrus MNI: x=-46 y=-62 z=50			
	**Df**	**F value**	**Pr(>F)**
FD	1,16	1.229	0.284
functional connectivity	1,16	11.434	0.004
MT by functional connectivity	1,16	0.545	0.471

l. precentral gyrus MNI: x=-44 y=0 z=54			
	**Df**	**F value**	**Pr(>F)**
FD	1,16	1.855	0.192
functional connectivity	1,16	26.101	0.000
MT by functional connectivity	1,16	0.127	0.726

An ANCOVA including the individual framewise displacement as first factor, 'mean functional connectivity' as second factor, and their interaction as third factor were computed. Using type I sums of squares, it was tested whether each factor led to an incremental improvement in error reduction. The results documented for all brain regions that the factor "mean functional connectivity" was significantly associated with the accuracy over and above possible effects of framewise displacement.

Supplement Tab. 3. *Effects of "TMS administration order" on the correlation between functional connectivity and TMS induced changes in accuracy detecting bilateral trials.*

l. superior temporal gyrus MNI: x=-48 y=0 z=-4			
	**Df**	**F value**	**Pr(>F)**
TMS administration	1,16	1.012	0.330
functional connectivity	1,16	26.537	0.000
TMS administration by functional connectivity	1,16	0.206	0.656

l. superior temporal gyrus MNI: x=-48 y=0 z=-2			
	**Df**	**F value**	**Pr(>F)**
TMS administration	1,16	1.061	0.318
functional connectivity	1,16	28.590	0.000
TMS administration by functional connectivity	1,16	0.253	0.622

l. angular gyrus MNI: x=-46 y=-62 z=50			
	**Df**	**F value**	**Pr(>F)**
TMS administration	1,16	0.955	0.343
functional connectivity	1,16	16.594	0.001
TMS administration by functional connectivity	1,16	7.754	0.013

l. precentral gyrus MNI: x=-44 y=0 z=54			
	**Df**	**F value**	**Pr(>F)**
TMS administration	1,16	1.071	0.316
functional connectivity	1,16	28.848	0.000
TMS administration by functional connectivity	1,16	0.417	0.528

The effect of TMS administration order ('TMS first, sham second' vs. 'Sham first, TMS second') on the linear association between functional connectivity and TMS-induced changes in the accuracy of bilateral targets were analyzed within an GLM. This ANCOVA included the order of TMS conditions as first factor, the mean functional connectivity as second factor, and their interaction as third factor. For each factor the incremental improvement in error reduction (type I sums of squares) was investigated. For all brain regions the linear association between mean functional connectivity following TMS and the TMS effects on the accuracy of bilateral trials showed a significant linear association over and above possible effects of the order of TMS condition.

Literature

1 Burnham, K. P., Anderson, D. R. & Burnham, K. P. *Model selection and multimodel inference : a practical information-theoretic approach*. 2nd edn, (Springer, 2002).

2 Okamoto, M. *et al.* Three-dimensional probabilistic anatomical cranio-cerebral correlation via the international 10–20 system oriented for transcranial functional brain mapping. *NeuroImage* **21**, 99-111, doi:10.1016/j.neuroimage.2003.08.026 (2004).

3 Yeo, B. T. *et al.* The organization of the human cerebral cortex estimated by intrinsic functional connectivity. *J. Neurophysiol.* **106**, 1125-1165, doi:10.1152/jn.00338.2011 (2011).

4 Buckner, R. L., Krienen, F. M., Castellanos, A., Diaz, J. C. & Yeo, B. T. The organization of the human cerebellum estimated by intrinsic functional connectivity. *J. Neurophysiol.* **106**, 2322-2345, doi:10.1152/jn.00339.2011 (2011).
